# SUMOylation of the polycomb group protein L3MBTL2 facilitates repression of its target genes

**DOI:** 10.1093/nar/gkt1317

**Published:** 2013-12-24

**Authors:** Christina Stielow, Bastian Stielow, Florian Finkernagel, Maren Scharfe, Michael Jarek, Guntram Suske

**Affiliations:** ^1^Institute of Molecular Biology and Tumor Research, Philipps-University, Emil-Mannkopff-Str. 2, D-35032 Marburg and ^2^Helmholtz Centre for Infection Research (HZI), Inhoffenstraße 7, D-38124 Braunschweig, Germany

## Abstract

Lethal(3) malignant brain tumour like 2 (L3MBTL2) is an integral component of the polycomb repressive complex 1.6 (PRC1.6) and has been implicated in transcriptional repression and chromatin compaction. Here, we show that L3MBTL2 is modified by SUMO2/3 at lysine residues 675 and 700 close to the C-terminus. SUMOylation of L3MBTL2 neither affected its repressive activity in reporter gene assays nor it’s binding to histone tails *in vitro*. In order to analyse whether SUMOylation affects binding of L3MBTL2 to chromatin, we performed ChIP-Seq analysis with chromatin of wild-type HEK293 cells and with chromatin of HEK293 cells stably expressing either FLAG-tagged SUMOylation-competent or SUMOylation-defective L3MBTL2. Wild-type FLAG-L3MBTL2 and the SUMOylation-defective FLAG-L3MBTL2 K675/700R mutant essentially occupied the same sites as endogenous L3MBTL2 suggesting that SUMOylation of L3MBTL2 does not affect chromatin binding. However, a subset of L3MBTL2-target genes, particularly those with low L3MBTL2 occupancy including pro-inflammatory genes, was de-repressed in cells expressing the FLAG-L3MBTL2 K675/700R mutant. Finally, we provide evidence that SUMOylation of L3MBTL2 facilitates repression of these PRC1.6-target genes by balancing the local H2Aub1 levels established by the ubiquitinating enzyme RING2 and the de-ubiquitinating PR–DUB complex.

## INTRODUCTION

Methylation of histone tails represents a crucial posttranslational modification involved in transcriptional regulation. Methylated histone tails are recognized by chromatin-reading modules such as Chromo, Tudor, PWWP and MBT (malignant brain tumor) domains, together designated as the ‘Royal family’ of chromatin binding domains (1). MBT-domain proteins contain arrays of two (2,3), three (4–6) or four (7–9) MBT domains that form an interlocked substructure that bind specifically to mono- and dimethylated lysine residues on histone tails. In humans, the family of MBT-domain proteins comprises nine members, which can be almost invariably linked to one of the three *Drosophila* MBT-domain proteins L(3)mbt, Scm and Sfmbt. All family members fulfil functions in differentiation, regulation of mitosis or tumor suppression (10).

Lethal(3) Malignant Brain Tumor Like 2 (L3MBTL2) represents the human ortholog of the *Drosophila* polycomb group protein Sfmbt. It possesses a zinc finger domain at the N-terminal part and four centrally located MBT domains, of which the most C-terminal one mediates binding to methyl groups *in vitro* (8). L3MBTL2 acts as a transcriptional repressor (11,12) and is involved in compaction of chromatin (12). Originally, L3MBTL2 was described as a subunit of the E2F6.com-1 complex in HeLa cells along with E2F6, MGA, MAX, DP1, HP1γ, G9a, GLP, RING1, RING2, PCGF6 and YAF2 (13). The majority of these proteins were also found to be associated with L3mbtl2 in murine embryonic stem cells (14). In addition, L3MBTL2 was identified as a crucial subunit of the PRC1 subcomplexes PRC1L4 (12) and polycomb repressive complex 1.6 (PRC1.6) (15). Genome-wide binding studies in K562 cells revealed a large overlap (>50%) between L3MBTL2- and E2F6-binding sites but no correlation with repressive histone marks (12). Consistently, full-length L3MBTL2 bound to histone tails and compacted nucleosomal arrays in a histone methylation-independent manner (12). The physiological relevance of L3mbtl2 was shown in mice, where it is essential for embryonic development (14). L3mbtl2 deficiency is embryonic lethal with failure in gastrulation. Moreover, proliferation of L3mbtl2^−^^/^^−^ embryonic-stem cells is strongly impaired due to a prolonged G_0/1_ phase (14).

Many proteins regulating gene expression including histones and chromatin-associated enzymes are reversibly modified by SUMO thereby affecting gene expression positively or negatively (16). SUMOylation occurs through an enzymatic cascade including a heterodimeric E1 enzyme (AOS1/UBA2), an E2 enzyme (UBC9) and an E3 ligase, the latter enhancing the rate of SUMOylation, and potentially contributing to specificity (17). Vertebrates express three functional SUMO paralogs (SUMO1, 2 and 3) (18) of which SUMO2 and SUMO3 are 97% identical and are therefore referred to as SUMO2/3. The covalent attachment of SUMO occurs mainly at lysine residues within the consensus sequence ΨKXE (19) but non-consensus SUMO-attachment sites are also known (20).

Here, we report the identification of L3MBTL2 as a novel SUMOylated protein that is specifically modified with SUMO2/3 at the two C-terminal lysine residues K675 and K700. SUMOylation of L3MBTL2 was not required for its repressive activity in reporter gene assays, its binding to histone tails *in vitro* or its site-specific recruitment to chromatin *in vivo*. However, we found that a subset of weakly occupied L3MBTL2-target genes including proinflammatory genes was de-repressed in cells expressing a SUMOylation-defective L3MBTL2 mutant. Hence, we conclude that SUMOylation facilitates repression of L3MBTL2-target genes following chromatin recruitment.

## MATERIALS AND METHODS

Antibodies used for immunodetection and ChIP experiments as well as experimental procedures for plasmid construction, reporter-gene assays, generation of stable cell lines, western blotting, immunoprecipitation and quantitative real-time PCR are described in ‘Materials and Methods’ section of the Supplementary Material.

### Nickel affinity purification

HEK293 cells were seeded at a density of 1 × 10^6^ cells per 10-cm dish, and 24 h after seeding transfected with either with 3 µg of L3MBTL2 expression vectors or 1.5 µg of L3MBTL2 expression vectors along with 1.5 µg of His-SUMO expression plasmids using FuGENE HD (Promega). Forty-eight hours post transfection, cells were lysed in 1 ml of 6 M guanidinium HCl, 0.1 M sodium phosphate buffer, pH 8.0, 0.05% Tween 20, 20 mM imidazole. His-SUMO modified proteins of transfected HEK293 cells or of HeLa cells stably expressing 6xHis-SUMO1, 6xHis-SUMO2 or 6xHis-SUMO3 (21) were isolated by incubation with 20 µl of Ni-NTA agarose or magnetic agarose beads (Qiagen) over night at 4°C. Beads were washed three times each with 750 µl of buffer A (8 M urea, 0.1 M sodium phosphate buffer, pH 8.0, 0.05% Tween 20, 20 mM imidazole) and buffer B (8 M urea, 0.1 M sodium phosphate buffer, pH 6.4, 0.05% Tween 20, 20 mM imidazole). After a final washing step with phosphate buffered saline, the beads were boiled in 2x SDS Laemmli buffer and analysed by western blotting.

### Knockdown of endogenous proteins

The siGenome Smart Pool (M-010678-00) and two individual OnTarget plus siRNAs (J-010678-17/-18) from Thermo Scientific Dharmacon were used for RNAi-mediated knockdown of human L3MBTL2. For depletion of RING2 (J-006556), ASXL1 (J-012856), BAP1 (J-005791) and PIAS1 (J-008167) pools of four individual OnTarget plus siRNAs were used. The siGenome non-targeting siRNA #1 (D-001210-01) and an siRNA targeting firefly luciferase (D-002050-01) were used as unspecific siRNA controls. HEK293 cells on six-well plate were transfected with 20 nM siRNA using OligofectamineTM (Invitrogen). Three days post-transfection 3 × 10^5^ cells were replated, and transfected a second time. Additional 3 days later, cells were collected and knockdown efficiency was monitored by RT-qPCR and western-blot analysis.

For depletion of endogenous SUMO isoforms, HEK293 cells were transiently transfected with shRNA expression constructs targeting SUMO1 or SUMO2/3. Following selection with 1 µg/ml of puromycin or 300 µg/ml of zeocin for 96 h, whole-cell extracts were prepared and analysed by western blotting.

### *In vitro* SUMOylation of L3MBTL2

Expression and purification of recombinant SUMO1, SUMO2, E1 (6xHis-AOS1/UBA2) and E2 enzyme (UBC9) were described (22,23). SUMO modification reactions of bacterially expressed 6xHis-L3MBTL2 was carried out at 37°C for the indicated periods in a total volume of 10 µl reaction buffer (20 mM HEPES/KOH, pH 7.3, 110 mM potassium acetate, 2 mM magnesium acetate, 0.5 mM EGTA, 0.05% Tween 20, 0.4 mg/ml ovalbumin and 0.5× protease inhibitor cocktail, Roche) containing ∼1 µg of 6xHis-L3MBTL2 protein, 3 ng/µl E1 enzyme (6xHis-AOS1/UBA2), 5 ng/µl E2 enzyme (UBC9), 10 ng/µl SUMO2 and 2 mM ATP. The effect of PIAS1 on L3MBTL2 SUMOylation was investigated by the addition of baculovirus-expressed 6xHis-PIAS1. Reactions were stopped either by adding 2× SDS Laemmli buffer or by 1 U of apyrase (Sigma) per micromoles of ATP.

### Peptide-binding assays

Five microgram of histone peptides (H3 residues 1–15, H4 residues 16–25) bearing C-terminal cysteine residues were immobilized on 40 µl of SulfoLink Coupling Resin (Thermo Scientific) in 100 µl of coupling buffer (50 mM Tris–HCl, pH 8.5, 5 mM EDTA) for 90 min at room temperature. Free iodoacetyl groups were blocked with 50 mM L-cysteine for 90 min at room temperature. Upon extensive washing with 1 M NaCl, beads were blocked with 50 µl bovine serum albumin (10 mg/ml) for 1 h at 4°C. Immobilized peptides were incubated on a rotating wheel with recombinant proteins in 1 ml of binding buffer (25 mM Tris–HCl, pH 8, 150 mM NaCl, 2 mM EDTA, 0.5% NP 40) for 1 h at 4°C. Beads were washed six times with binding buffer, and bound proteins were analysed by western blotting.

### ChIP-qPCR and ChIP-Seq

ChIP-qPCR and ChIP-Seq experiments were performed as described previously (24,25) using the OneDay ChIP kit (Diagenode) in accordance to the manufacturer’s instructions. The gene-specific primers used for ChIP-qPCRs are listed in Materials and Methods section of Supplementary Material.

### ChIP-Seq data analysis

Raw ChIP-Seq data were aligned to the human genome assembly GrCh37 (hg19) with Bowtie version 0.12.7 allowing for two mismatches in the seed and a total mismatch quality sum score of 70 (–*n* 2 –*e* 70). Reads mapping to multiple locations on the genome were discarded (–*m* 1 –*k* 1), and Bowtie output was converted to BAM format. Reads were filtered to a maximum of two reads per start position and start positions with more than seven reads were censored. The following amounts of usable, uniquely matching reads were obtained: HEK293 cells, anti L3MBTL2: 19 660 936; HEK293 cells, anti FLAG: 26 605 142; HEK293 cells, anti IgG: 22 346 403; HEK293 expressing 3xFLAG L3MBTL2 WT, anti FLAG: 22 064 463; HEK293 expressing 3xFLAG L3MBTL2 K675/700, anti FLAG: 20 599 800.

Peak calling was performed with MACS version 1.4.0rc2 20110214 modified for reading BAM files via pysam (http://code.google.com/p/pysam/), and peaks with >100 tags in the IgG control or the anti FLAG control were excluded. This yielded 41 570 (HEK293, anti L3MBTL2), 37 846 (HEK293, 3xFLAG L3MBTL2 WT, anti FLAG) and 44 781 (HEK293, 3xFLAG L3MBTL2 K675/700, anti FLAG) putative L3MBTL2-binding sites, of which 8009, 14 986 and 9664, respectively, had a MACS defined false discovery rate (FDR) of ≤0.001.

Genes- and L3MBTL2-binding sites were associated by testing for overlap between peak regions and transcription start sites ±1250 bp. Assignment of homology between human and mouse L3MBTL2-bound genes is based on the following procedure. Genes were defined as L3MBTL2-bound when they had a binding site within ±10 kb of a transcription start site (for mES cells, only a list of such binding sites was available). The resulting gene sets were paired via a one-to-one bi-directional relationship in Ensembl Compara, and a Venn diagram depicting whether the pair was L3MBTL2-bound in HEK293 cells, mES cells or both was drawn. Genes that were L3MBTL2-bound but did not have a one-to-one correspondence with the other species (species-specific genes) were included as separate circles.

RNA polymerase II and H3K4 ChIP-Seq were retrieved from the UCSC genome browser (track names: wgEncodeSydhTfbsHek293Pol2StdAlnRep3/wgEncodeUwHistoneHek293H3k4me3StdPkRep1, Gene Expression Omnibus accession GSM935534/GSM945288). Both datasets were used in pre-processed form.

Motif search was performed with MEME-ChIP version 4.9.0 and Centrimo (26,27) using all sequences surrounding L3MBTL2 peak summits (±150 bp). Summits were defined by pooling L3MBTL2 reads from all three L3MBTL2 ChIP experiments elongating them to 200 bp and determining the position of highest overlap.

### Pathway analysis

Functional enrichment analysis was performed with ChIP-Seq-derived gene lists versus predefined gene sets using Fisher’s exact test. The Benjamini–Hochberg (28) procedure was used to correct for multiple hypothesis testing.

### Databases and data deposition

Genome sequences, annotation and homology data were retrieved from Ensembl revision 65 (http://dec2011.archive.ensembl.org). Known transcription factor motifs were retrieved from Jaspar, UniProbe and Matbase (Genomatix). Gene sets from the PANTHERDB version 7.0 were used for pathway analysis (29). The ChIP-Seq data from this publication have been submitted to the ArrayExpress and the European Nucleotide Archive database (http://www.ebi.ac.uk/arrayexpress/, http://www.ebi.ac.uk/ena/home) and assigned the identifier E-MTAB-1731 and ERP003468, respectively.

## RESULTS

### L3MBTL2 is a SUMOylated protein

**Figure 1.**
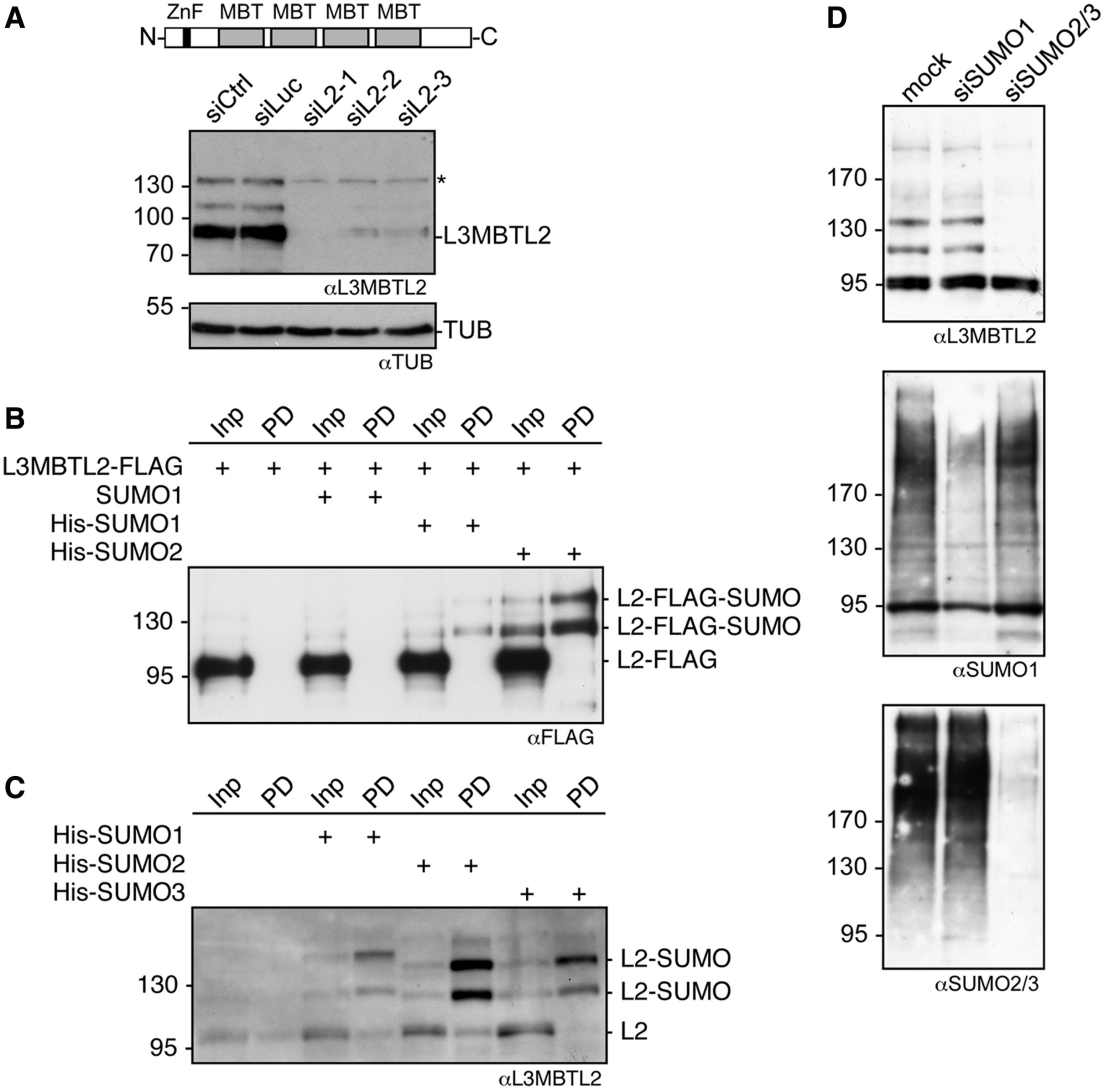
L3MBTL2 is a target for SUMOylation. (**A**) Top, schematic presentation of human L3MBTL2. MBT domains are shown in grey, the C_2_/C_2_-zinc finger in black. Bottom, depletion of endogenous L3MBTL2. HEK293 cells were treated with a non-targeting control siRNA (siCtrl), a luciferase-targeting siRNA (siLuc), a pool of four L3MBTL2 siRNAs (siL2-1) or two distinct L3MBTL2 siRNAs (siL2-2 and siL2-3). L3MBTL2 protein levels in whole-cell extracts were analysed by western blotting. Re-probing for Tubulin served as a loading control. The asterisk denotes a cross-reacting protein that co-localises with the upper L3MBTL2-specific signal. (**B**) L3MBTL2-FLAG was transfected along with untagged SUMO1, His-tagged SUMO1 or His-tagged SUMO2 into HEK293 cells. His-SUMO-conjugated proteins were subsequently purified from cell lysates by Ni-NTA affinity chromatography (Ni-pulldown, PD). SUMOylated L3MBTL2-FLAG was detected by immunoblotting for the FLAG-tag. Input: 10%, pulldown (PD): 45%. (**C**) His-SUMO-conjugated proteins were purified by Ni-pulldown from HeLa cells stably expressing His-SUMO1, His-SUMO2 or His-SUMO3. Endogenous L3MBTL2 was subsequently detected by western blotting. Input: 10%, pulldown (PD): 45%. (**D**) HEK293 cells were transfected with shRNA expression constructs targeting *SUMO1* or *SUMO2/3* mRNA. Subsequently, whole-cell extracts were analysed for L3MBTL2, SUMO1 and SUMO2/3 protein levels by western blotting.

Western-blot analysis of L3MBTL2 detected three protein bands, one running at the expected size of ∼95 kDa and two slower migrating forms (Figure 1A). All three protein bands are related to L3MBTL2 since RNAi-mediated knockdown of L3MBTL2 reduced the intensity of all three signals (Figure 1A). We hypothesized that the two high molecular weight L3MBTL2 species reflect covalent modification of L3MBTL2 by SUMO. To test whether L3MBTL2 could be SUMOylated *in vivo*, we transiently transfected FLAG-tagged L3MBTL2 along with His-tagged SUMO1 or His-tagged SUMO2 in HEK293 cells. Purification of His-SUMO conjugates under denaturing conditions followed by western blotting for FLAG-L3MBTL2 retrieved two slow migrating L3MBTL2 species (Figure 1B). L3MBTL2 was preferentially modified by His-SUMO2 although His-SUMO1 modified L3MBTL2 was detectable as well. The absence of any recovered FLAG-L3MBTL2 upon transfection of untagged SUMO1 (Figure 1B, lane 4) confirmed specificity of the L3MBTL2-His-SUMO signals. We also employed HeLa cells stably expressing either His-SUMO1, His-SUMO2 or His-SUMO3 (21). In these cells, endogenous L3MBTL2 was modified by all three His-SUMO isoforms with a preference for His-SUMO2 (Figure 1C). Finally, we knocked down endogenous SUMO1 or SUMO2/3 by SUMO-isoform specific shRNAs followed by western-blot analysis of L3MBTL2. The SUMO2/3 knockdown but not the SUMO1 knockdown resulted in a specific and almost complete loss of the two slow-migrating L3MBTL2-protein species (Figure 1D). Collectively, these results demonstrated that L3MBTL2 is a SUMOylated protein that is specifically modified by SUMO2/3 *in vivo*.

### L3MBTL2 is specifically SUMOylated at K675 and K700

**Figure 2.**
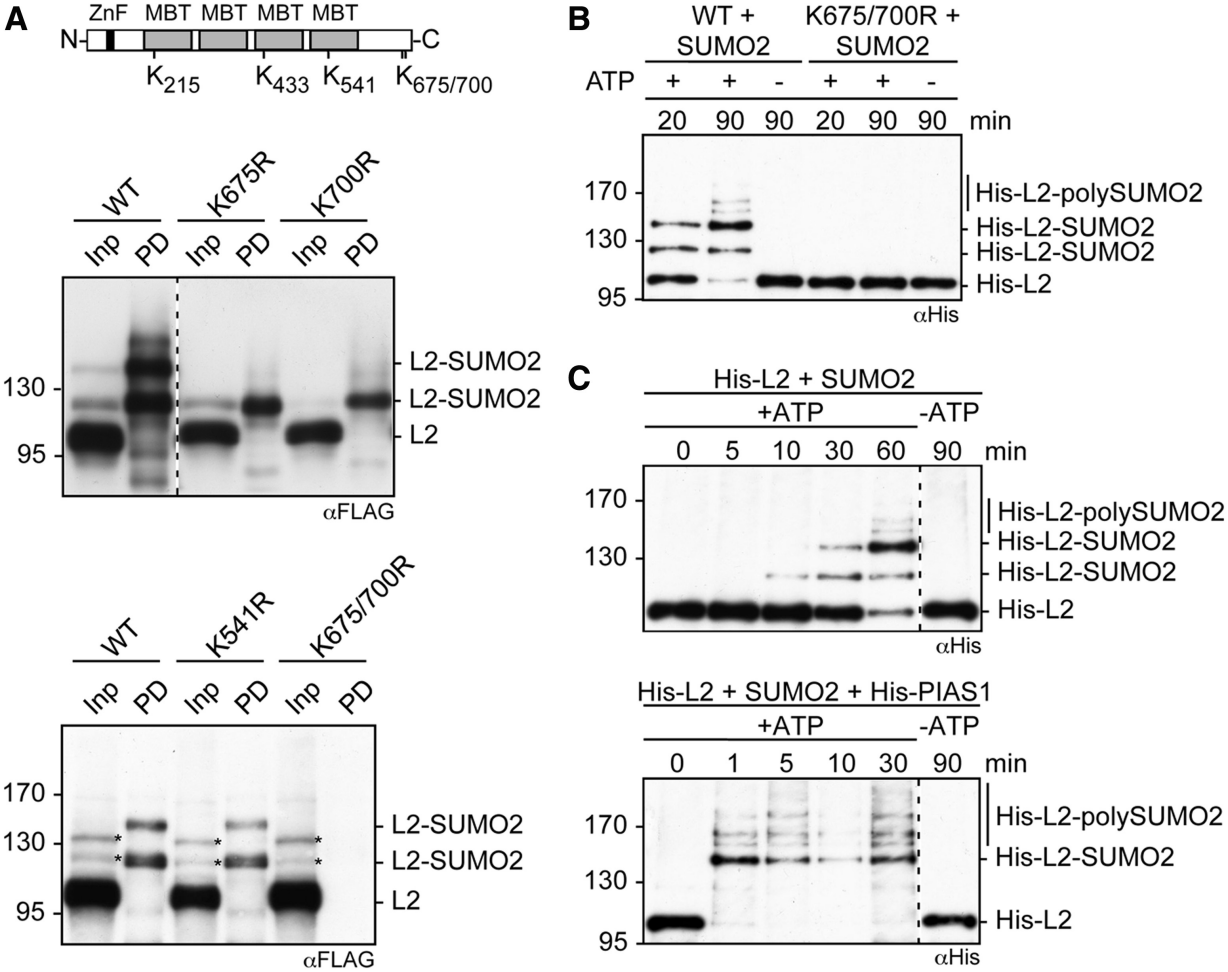
L3MBTL2 is SUMO-modified at lysines 675 and 700. (**A**) Top, position of lysine residues embedded in classical SUMO consensus sites (ΨKXE). Bottom, mutational analysis of potential SUMO acceptor lysines. The L3MBTL2-FLAG mutants K541R, K675R, K700R and K675/700R were expressed together with His-SUMO2 in HEK293 cells. His-SUMO2 conjugates were subsequently purified by Ni-pulldown and analysed for SUMOylated L3MBTL2-FLAG by western blotting using anti-FLAG antibodies. Input: 10%, pulldown (PD): 22.5%. The signals in the lower panel marked by asterisks were likely derived from cross-reacting proteins. (**B**) *In vitro* SUMOylation of L3MBTL2. Recombinant His-tagged wild-type L3MBTL2 or the L3MBTL2 K675/700R mutant was incubated with purified, recombinant E1 enzyme (AOS1/UBA2), E2 enzyme (UBC9) and SUMO2 in the presence or absence of ATP for the indicated time periods. The SUMOylation reactions were monitored by western blotting for His-L3MBTL2. (**C**) PIAS1 acts as an E3 ligase for L3MBTL2 SUMOylation *in vitro*. *In vitro* SUMOylation of wild-type L3MBTL2 was carried out as in (B) for the indicated time periods in the absence (top panel) or presence (bottom panel) of PIAS1 as indicated.

L3MBTL2 contains five perfect SUMOylation consensus motifs (ψKXE, with ψ representing a hydrophobic amino acid) at K215, K433 and K541 within the first, third and fourth MBT domains, respectively, and at K675 and K700 close to the C-terminus (Figure 2A). We analysed FLAG-L3MBTL2 mutants in which lysine residues at positions 541, 675 and 700 were replaced individually or in combination by arginines for SUMOylation by His-SUMO2 (Figure 2A). The His-SUMO2 modification pattern of the recovered L3MBTL2 K541R mutant after Ni-NTA agarose pulldown was similar to wild-type L3MBTL2 (compare lane 2 with lane 4 in Figure 2A, lower panel). In contrast, only one L3MBTL2-His-SUMO2 signal was detectable upon transfection of the L3MBTL2 K675R or the L3MBTL2 K700R mutant (Figure 2A, upper panel). The L3MBTL2 K675/700R double mutant lacked any SUMOylation signal (Figure 2A, lower panel) strongly suggesting that L3MBTL2 is specifically and exclusively SUMOylated at K675 and K700.

To further confirm specific SUMOylation of L3MBTL2 at K675 and K700, we performed an *in vitro* SUMOylation assay using purified recombinant E1 (AOS1 and UBA2 dimer) and E2 (UBC9) enzymes along with recombinant SUMO2 and His-L3MBTL2 or the His-L3MBTL2 K675/700R mutant as substrates. Wild-type L3MBTL2 but not the L3MBTL2 K675/700R mutant was efficiently SUMO-modified *in vitro* (Figure 2B). Taken together, our results demonstrate that L3MBTL2 is exclusively SUMOylated at K675 and K700.

Finally, we asked whether PIAS1 could act as an E3 ligase for SUMOylation of L3MBTL2. In brief, the addition of purified recombinant PIAS1 drastically enhanced the rate of SUMOylation (compare Figure 2C upper panel with lower panel) resulting in complete SUMOylation of L3MBTL2 already after a 1-min incubation. We also addressed whether PIAS1 does act as an E3 ligase for L3MBTL2 SUMOylation *in vivo* by treating HEK293 cells with *PIAS1* siRNAs. RNAi-mediated knockdown of PIAS1 did not reduce SUMOylation of endogenous L3MBTL2 (Supplementary Figure S1). Whether some residual PIAS1 activity was sufficient to SUMOylate L3MBTL2 or whether other SUMO E3 ligases catalyze SUMOylation of L3MBTL2 *in vivo* remains to be established.

### The L3MBTL2 K675/700R mutant retains repression activity in reporter gene assays

**Figure 3.**
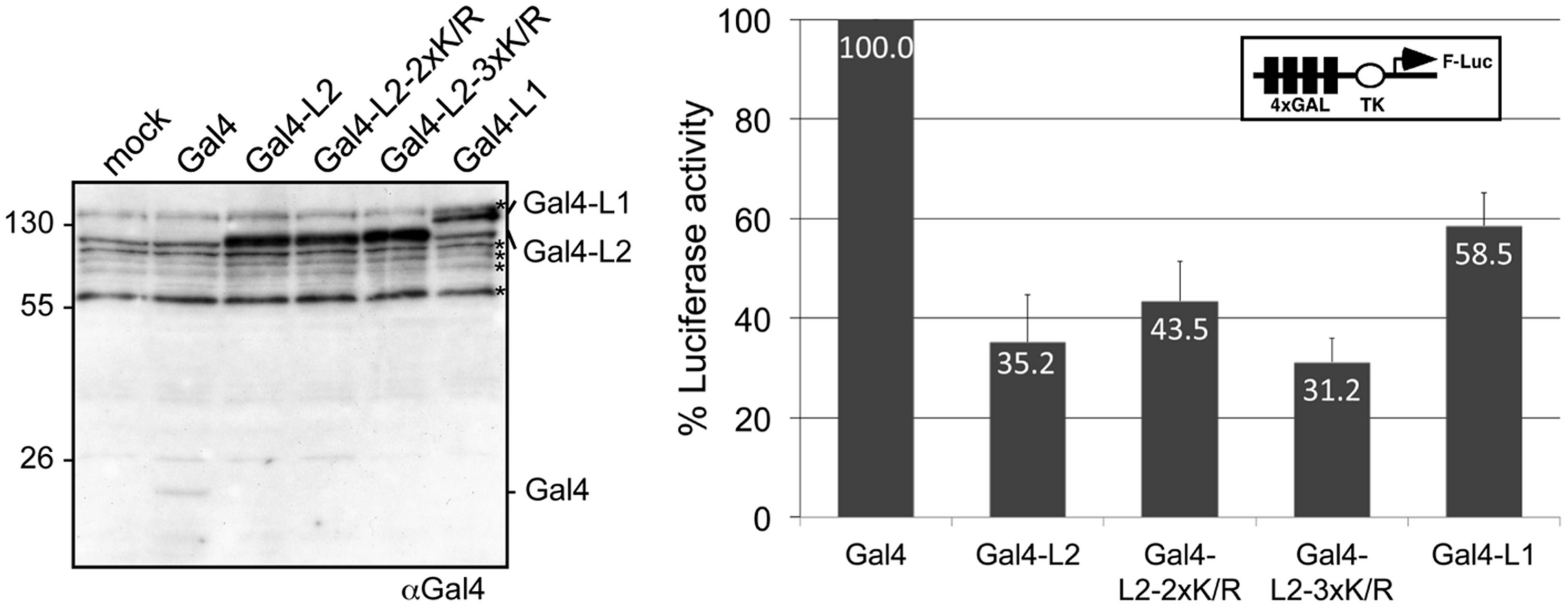
The L3MBTL2 K675/700R mutant retains repression activity in a reporter gene assay. HEK293 cells were transiently transfected with constructs expressing Gal4 (aa 1–147), Gal4-L3MBTL2 (Gal4-L2), Gal4-L3MBTL2 K675/700R (Gal4-L2-2xK/R), Gal4-L3MBTL2 K541/675/700R (Gal4-L2-3xK/R) or Gal4-L3MBTL1 (Gal4-L1), respectively, along with the 4xGal-TK-Luc reporter and a *Renilla* luciferase control reporter. Subsequently, whole-cell extracts were analysed for Gal4 expression (left panel) and luciferase activity (right panel). The reporter activity in the presence of Gal4 was set to 100%. Data are represented as mean ± SD of three independent experiments each performed in duplicate. The asterisks in the western-blot panel denote cross-reacting proteins.

Previously, it was shown that L3MBTL1, a close relative to L3MBTL2, fused to the Gal4 DNA-binding domain confers repression of a Gal4-responsive reporter gene (30). We asked whether L3MBTL2 could also act as a transcription repressor in such an assay and whether SUMOylation would mediate repression. Wild-type L3MBTL2 and the L3MBTL2 K675/700R mutant were fused to the Gal4 DNA-binding domain and transfected into HEK293 cells along with a Gal4-responsive UAS-TK-luciferase reporter plasmid. Gal4-L3MBTL1 was included as a positive control. The Gal4-L3MBTL1/2 proteins were expressed at similar levels (Figure 3A). Compared to the Gal4 DNA-binding domain on its own, Gal4-L3MBTL1, Gal4-L3MBTL2 and the Gal4-L3MBTL2 K675/700R mutant version repressed luciferase expression by 2- to 3-fold (Figure 3B). This result shows that L3MBTL2 can act as a repressor when tethered to a promoter by the Gal4 DNA-binding domain. However, SUMOylation appears to be dispensable for the repression activity in this assay.

### SUMOylated L3MBTL2 retains binding activity to histone tails *in vitro*

**Figure 4.**
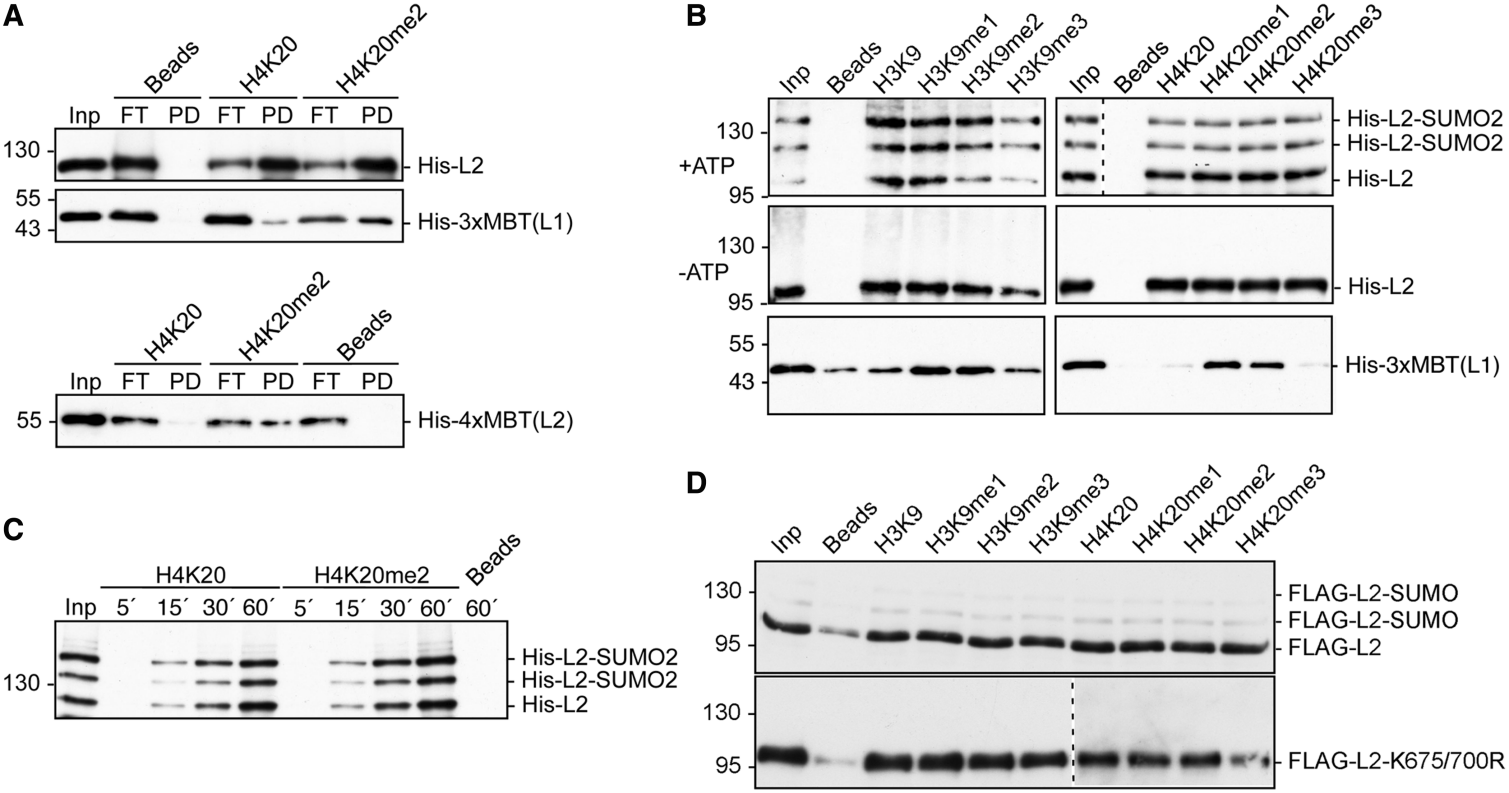
Binding of unmodified and SUMOylated L3MBTL2 to histone H3 and H4 tails. (**A**) Peptide binding of recombinant L3MBTL2. Immobilized H4K20 and H4K20me2 peptides (residues 16–25) were incubated either with recombinant full-length His-L3MBTL2 (His-L2), 3xMBT-repeat domain of L3MBTL1 (His-3xMBT-L1) or the 4xMBT-repeat domain of L3MBTL2 (His-4xMBT, aa 170–619). Binding of His-L3MBTL2 was analysed by western blotting using anti-His antibodies. Inp, input: 10%, FT, flow through: 20% (upper panels) or 10% (lower panel), PD, pull-down: bound material. (**B**) Full-length His-L3MBTL2 was SUMOylated *in vitro* and subsequently incubated with N-terminal histone H3 (residues 1–15) or H4 peptides (residues 16–25) containing the indicated methylated lysines. The 3xMBT-repeat domain of L3MBTL1 served as a positive control for specific binding to mono- and di-methylated histone peptides. Detection of bound His-L3MBTL1/2 was by western blotting using anti-His antibodies. Input: 10%. (**C**) Time course of peptide binding of unmodified and SUMOylated L3MBTL2. *In vitro* SUMOylated full-length His-L3MBTL2 was incubated with unmethylated (H4K20) or di-methylated H4 (H4K20me2) peptides for 5, 15, 30 and 60 min as indicated. Detection of bound His-L3MBTL2 was by western blotting using anti-His antibodies. Input: 10%. (**D**) Wild-type 3xFLAG-L3MBTL2 and the 3xFLAG-L3MBTL2 K675/700R mutant were immunoprecipitated from N-ethylmaleimide-treated nuclear extracts of stably transfected HEK293 cells and subsequently incubated with the indicated histone H3 and H4 peptides. Detection of bound FLAG-L3MBTL2 was by western blotting using anti-FLAG antibodies. Input: 10%.

Isolated MBT domain arrays including the 3xMBT-repeat domain of L3MBTL1 (4–6) and the 4xMBT-repeat domain of L3MBTL2 (8) bind *in vitro* to a number of histone tail peptides with a preference for mono- or di-methylated lysines. To analyse whether SUMOylation of L3MBTL2 affects binding to histone tails, we performed comprehensive binding studies with immobilized unmethylated and methylated peptides that correspond to N-terminal regions of histone H3 and histone H4 (Figure 4). Consistent with previous reports, the isolated 3xMBT-repeat domain of L3MBTL1 as well as the isolated 4xMBT-repeat domain of L3MBTL2 bound preferentially to a histone H4 peptide di-methylated at K20 as compared to the unmethylated peptide (Figure 4A). Full-length L3MBTL2, however, bound to the H4 peptide regardless of H4K20 di-methylation (Figure 4A). The absence of any significant binding preference of full-length L3MBTL2 for the dimethylated H4 histone tail is in line with a recent report (12). Next, we asked whether SUMOylation of L3MBTL2 affects binding to histone tails. We SUMOylated L3MBTL2 *in vitro* in such a way that similar amounts of unmodified and SUMO-modified L3MBTL2 were present in the peptide binding reactions. SUMOylated L3MBTL2 retained the ability to bind H3 and H4 histone peptides irrespectively of mono-, di- or tri-methylation at H3K9 or H4K20 (Figure 4B). Moreover, a time course experiment of L3MBTL2-binding to H4K20 and H4K20me2 revealed that SUMO modification of L3MBTL2 did also not alter the rate of binding (Figure 4C). Finally, we addressed whether L3MBTL2-associated proteins or additional post-translational modifications affect binding of L3MBTL2 to histone tail peptides. To this end we immunoprecipitated cellular L3MBTL2 from HEK293 cells that express either wild-type FLAG-L3MBTL2 or the FLAG-L3MBTL2 K675/700R mutant (see below). Wild type and mutant FLAG-L3MBTL2 bound H3 and H4 histone peptides irrespectively of whether they were un-, mono-, di- or tri-methylated (Figure 4D). In conclusion, SUMOylation of recombinant L3MBTL2 as well as cellular L3MBTL2 does not affect binding to histone tails *in vitro.*

### Genome-wide identification of L3MBTL2-binding sites in HEK293 cells

**Figure 5.**
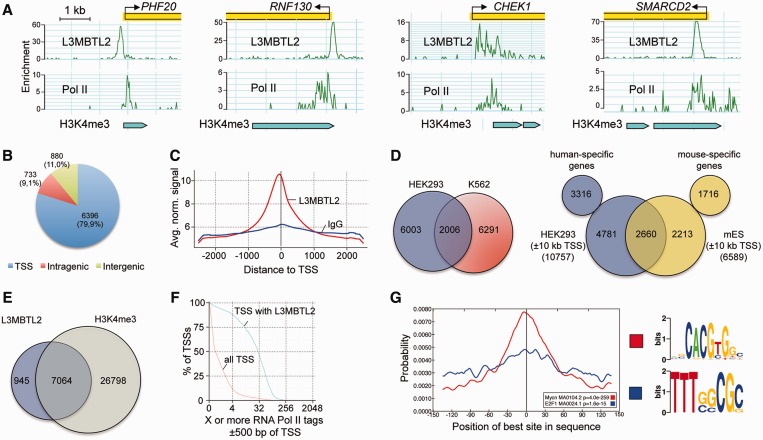
Overview of L3MBTL2 chromatin occupancy in HEK293 cells. (**A**) Examples of normalized HEK293 ChIP-Seq signals along with RNA polymerase II signals and H3K4me3 beds illustrate their co-occurrence at transcriptional start sites (TSSs). The plots show normalized ChIP-Seq signals for L3MBTL2, RNA polymerase II (GSM935534) and H3K4me3 beds (GSM945288) at the *PHF20*, *RNF130*, *CHEK1* and *SMARCD2* genes. (**B**) Distribution of L3MBTL2 occupancy relative to annotated genes. (**C**) Averaged L3MBTL2 coverage around TSSs in HEK293 cells normalized to 20 million reads. (**D**) Venn diagrams illustrating the overlap of L3MBTL2-binding sites between HEK293 and K562 (12) cells (left), and of L3MBT2 bound genes between HEK293 and mouse ES cells (14) (right). For HEK293/mES comparison, L3MBTL2 signals at ±10 kb of TSSs were used to assign bound genes (see Materials and methods section for details), because only a list of L3MBTL2 peaks ±10 kb was available for mouse ES cells. (**E**) Venn diagram illustrating the overlap between L3MBTL2 binding sites and the H3K4me3 signature (GSM945288) in HEK293 cells. (**F**) RNA polymerase II is enriched at TSSs with L3MBTL2 peaks. All TSSs (red line) and TSSs bound by L3MBTL2 (blue line) were plotted against RNA Pol II ChIP-Seq tag counts (GSM935534). (**G**) Centrally enriched sequence motifs at L3MBTL2-binding sites obtained by running Centrimo (27) with 300 bp summits of all 8006 FDR ≤ 0.001 L3MBTL2 sites.

We sought to analyse whether SUMOylation of L3MBTL2 affects chromatin occupancy or target gene expression. As a prerequisite we carried out genome-wide chromatin immunoprecipitation followed by sequencing (ChIP-Seq) for L3MBTL2 in HEK293 cells. In brief, using an FDR of ≤0.001, MACS peak calling (31) identified a total of 8009 high confidence peaks (Supplementary Dataset S1). Snapshots of representative L3MBTL2-binding sites are shown in Figure 5A, top panels. The large majority of the L3MBTL2-binding sites (79.9%) were located in the proximity of the 5′-end (±1250 bp) of annotated transcripts (Figure 5B and C) encompassing 7764 genes. Roughly 11% and 9% of the binding sites were intergenic or intragenic, respectively (Figure 5B). ChIP-Seq analysis of L3MBTL2 has recently also been performed with chromatin of human K562 cells (12) and mouse embryonic stem cells (14). Approximately one third of the L3MBTL2 binding sites in HEK293 cells overlap with the reported sites in K562 cells and in mouse embryonic stem cells (Figure 5D). The partial overlap of L3MBTL2-binding sites in different cell types of different species indicates that L3MBTL2 regulates general as well as cell-type specific processes.

Comparison of L3MBTL2-binding sites with promoter regions containing H3K4me3 marks (Figure 5E), and with sites occupied by RNA polymerase II in HEK293 cells (Figure 5F) (32) revealed that the majority of L3MBTL2 is bound to promoters of active genes or of repressed genes that are poised for transcription. We used MEME-ChIP (26), including Centrimo (27), for DNA motif search at the centre of the L3MBTL2 peaks. Most significantly, Centrimo identified the E-box with the CACGTG core sequence, which represents a binding site for members of the MYC transcription factor family, as the most prevalent centrally enriched motif (Figure 5G). Interestingly, the motif known to be bound by E2F6, which is associated with L3MBTL2 in the E2F6.com-1 complex (13) and the PRC1.6 complex (15), occurred with much less probability and is barely enriched at the peak centres of the L3MBTL2 binding sites. Finally, we examined the biological processes and molecular functions associated with L3MBTL2-target genes. PANTHER gene ontology terms were primarily enriched for nucleotide-involving processes such as ‘mRNA transcription’, ‘nuclear mRNA splicing, via spliceosome’, ‘DNA repair’ and ‘DNA replication’. Of particular note is the large number of transcription factor genes, which led to enrichment of the categories ‘GO:0003700 transcription factor activity’, ‘PC00218 transcription factor’, ‘PC00244 zinc finger transcription factor’ and ‘PC00029 KRAB box transcription factor’. In total 18% of all L3MBTL2-bound and functionally annotated genes fall in these categories (Supplementary Figure S2).

### Ectopically expressed L3MBTL2 and the L3MBTL2 K675/700R mutant retain binding to endogenous L3MBTL2-target genes

**Figure 6.**
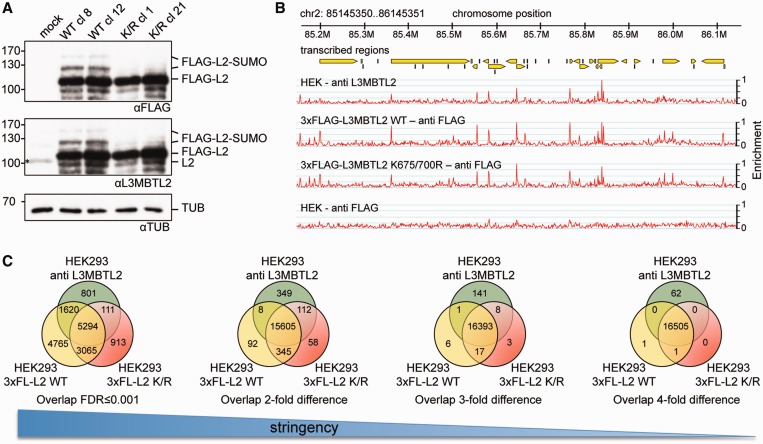
Ectopically expressed wild-type L3MBTL2 and the SUMOylation-defective L3MBTL2 K675/700R mutant retain specific binding to L3MBTL2 target genes. (**A**) Western-blot analysis of stably transfected HEK293 cells expressing either 3xFLAG wild type L3MBTL2 (WT) or the 3xFLAG L3MBTL2 K675/700R mutant (K/R). Single clones were lysed in SDS-containing buffer and analysed for L3MBTL2 expression using either anti-FLAG- or anti-L3MBTL2-specific antibodies as indicated. Two representative wild type (WT cl 8 and 12) and mutant clones (K/R cl 1 and 21) are shown. Anti-tubulin staining served as a loading control. The asterisk in the middle panel of the mock lane denotes endogenous L3MBTL2. (**B**) Genome browser snapshots of ChIP-Seq patterns of endogenous L3MBTL2 (HEK—anti L3MBTL2) and ectopically expressed 3xFLAG-L3MBTL2 WT and 3xFLAG-L3MBTL2 K675/700R for a region on chromosome 2. ChIP-Seq with chromatin from parental HEK293 cells using an anti-FLAG antibody (HEK - anti FLAG, bottom panel) served as a negative control. Genomic coordinates and transcribed regions according to ENSEMBLE (yellow arrows) are indicated at the top. (**C**) Venn diagrams illustrating the overlap between endogenous L3MBTL2, 3xFLAG-L3MBTL2 WT and 3xFLAG-L3MBTL2 K675/700R-binding sites under different stringent filtering conditions. Left Venn diagram: overlap of binding sites with an FDR ≤ 0.001. The three Venn diagrams on the right side were obtained by merging the FDR ≤ 0.001 sites of all three ChIP-Seq datasets and subsequent comparison allowing for a 2-, 3- or 4-fold difference in read counts at individual peaks (see ‘Materials and Methods’ section for details).

To assess whether SUMOylation affects chromatin occupancy of L3MBTL2, we generated HEK293 cells stably expressing either 3xFLAG-tagged wild-type L3MBTL2 or the 3xFLAG-tagged L3MBTL2 K675/700R mutant. Consistent with the assignment of K675 and K700 as the SUMO-target sites, FLAG-tagged wild-type L3MBTL2 but not the L3MBTL2 K675/700R mutant was SUMOylated (Figure 6A). Single clones that express SUMOylated wild-type L3MBTL2 or the SUMOylation-defective L3MBTL2 K675/700R mutant at similar level (clones number 8 and 21 in Figure 6A) were selected for further analysis.

To explore whether ectopically expressed FLAG-L3MBTL2 occupied the same sites as endogenous L3MBTL2 we performed ChIP-Seq using anti-FLAG antibodies. Figure 6B shows enrichment peaks of endogenous L3MBTL2, FLAG-L3MBTL2 WT and FLAG-L3MBTL2 K675/700R on chromosome 2 illustrating very similar enrichment patterns of all three proteins. Bioinformatics analysis using an FDR ≤ 0.001 identified 14 986 peaks for FLAG-tagged wild-type L3MBTL2 and 9664 peaks for the L3MBTL2 K675/700R mutant (Supplementary Dataset S1). Merging these two datasets with the dataset of endogenous L3MBTL2 revealed a strong overlap (Figure 6C, Venn diagram on the left), but at first sight also suggested the presence of wild-type L3MBTL2- and of L3MBTL2 K675/700R-specific binding sites. However, ChIP-qPCR experiments on selected promoters did not confirm any preferential binding of endogenous L3MBTL2, FLAG-L3MBTL2 WT or the FLAG-L3MBTL2 K675/700R mutant as exemplified by the *UGGT2*, *PRAF2* or *DENND3* promoters (Supplementary Figure S3) that were present in the dataset of endogenous L3MBTL2 and FLAG-L3MBTL2 WT but not in the dataset of the L3MBTL2 K675/700R mutant. The absence of a listed peak in one of the three datasets could also be due to the high stringency threshold of FDR ≤ 0.001 that we used for initial peak selection. Therefore, we applied less stringent conditions for dataset comparison. Merging all peaks with an FDR ≤ 0.001 in any of the three L3MBTL2 datasets yielded 16 569 peaks. Requiring a 2-, 3- or 4-fold difference in tag counts of individual L3MBTL2 peaks revealed a progressive greater overlap of the three ChIP-Seq datasets (Figure 6C) resulting in an almost complete overlap when tolerating a 4-fold difference in tag counts. On that score we concluded that binding of ectopically expressed wild-type FLAG-L3MBTL2 and the FLAG-L3MBTL2 K675/700R mutant mirrored binding of endogenous L3MBTL2 and did not result in additional ‘artificial’ chromatin recruitment.

**Figure 7.**
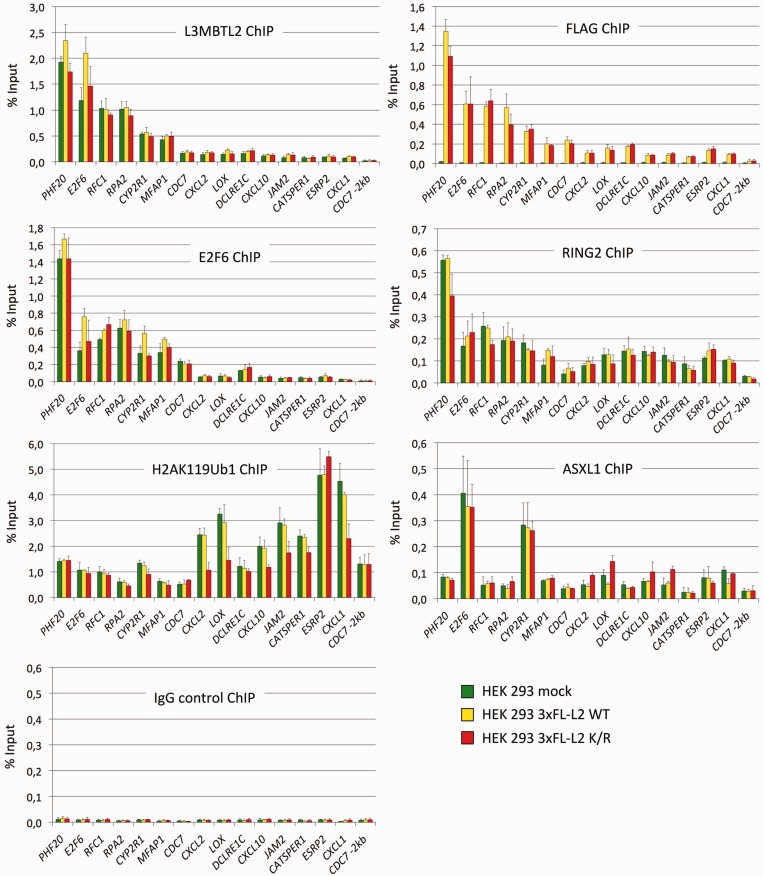
ChIP-qPCR analysis of L3MBTL2 (L3MBTL2 ChIP and FLAG ChIP), E2F6, RING2, H2AK119ub1 and ASXL1 at selected L3MBTL2-bound regions genes in mock-transfected HEK293 cells (green bars) and in HEK293 cells expressing 3xFLAG-L3MBTL2 WT (yellow bars) or the 3xFLAG-L3MBTL2 K675/700R mutant (red bars). The percent of input values are mean ±SD of at least three independent experiments. A list of gene abbreviations is found in Supplementary Table S1.

Given the striking similarity of ChIP-Seq peaks obtained for endogenous L3MBTL2 and ectopically expressed FLAG-L3MBTL2 WT and the FLAG-L3MBTL2 K675/700R mutant, we probed a panel of selected target genes by ChIP-qPCR using anti L3MBTL2 and anti FLAG antibodies (Figure 7, top panels). These experiments confirmed binding of L3MBTL2 at promoters with high read counts such as *PHF20*, *E2F6*, *RFC1* and *RPA2* as well as those with low read counts including *CXCL2*, *LOX*, *JAM2*, *CATSPER1* and *ESRP2*. Remarkably, over-expression of FLAG-L3MBTL2 WT and FLAG-L3MBTL2 K675/700R did not result in increased L3MBTL2 occupancy at these target sites either at promoters with high read counts or at promoters with low read counts suggesting that L3MBTL2 recruitment is saturated at physiological conditions. Importantly, this result also implied that SUMOylation competent FLAG-L3MBTL2 WT and the SUMOylation-defective FLAG-L3MBTL2 K675/700R largely replaced endogenous L3MBTL2. Hence, we concluded that SUMOylation of L3MBTL2 does not affect recruitment to target sites *in vivo*.

### E2F6, RING2, H2AK119ub1 and ASXL1 are present at L3MBTL2- target sites

L3MBTL2 is present in complexes that contain E2F6 and the ubiquitin E3 ligase RING2 (12–15). To assess whether over-expression of FLAG-L3MBTL2 or FLAG-L3MBTL2 K675/700R affected recruitment of these proteins, we also performed ChIP-qPCR analysis of E2F6 and RING2 on the selected panel of L3MBTL2 target genes. Both proteins were present at the L3MBTL2 sites regardless of FLAG-L3MBTL2 or FLAG-L3MBTL2 K675/700R over-expression (Figure 7). This result indicates that SUMOylation of L3MBTL2 does also not affect the recruitment of the entire L3MBTL2-containing PRC1.6 complex to its target sites. Finally, we analysed for the presence of mono-ubiquitinated H2A (H2AK119ub1) catalysed by RING2. The H2AK119ub1 mark was found at all promoters. Strikingly, however, the amount of H2AK119ub1 did not directly correlate but rather inversely correlated with the amount of L3MBTL2, E2F6 and RING2 (Figure 7). Promoters with relatively high L3MBTL2 and E2F6 levels such as *PHF20* and *E2F6* contained significantly lower levels of H2AK119ub1 than promoters with low L3MBTL2 and E2F6 levels such as *ESRP2* and *CXCL1*. Remarkably, at several weakly occupied L3MBTL2 target promoters (*CXCL2*, *LOX*, *CXCL10*, *JAM2*, *CATSPER1* and *CXCL1*), the H2AK119ub1 mark was specifically and reproducibly reduced up to 2-fold in cells expressing the FLAG-L3MBTL2 K675/700R mutant (Figure 7) suggesting that SUMOylation of L3MBTL2 facilitates mono-ubiquitination of H2AK119 or, alternatively, impedes deubiquitination of H2AK119ub1 at these promoters.

Since RING2 occupancy was unchanged at promoters that showed reduced H2AK119 ubiquitination (Figure 7) in cells expressing the L3MBTL2 K675/700R mutant, we also analysed for the presence of deubiquitinases. Several mammalian H2A deubiquitinating enzymes (DUBs) have been reported including 2A-DUB/Mysm1 (33), USP16/Ubp-M (34) and the dimeric polycomb repressive deubiquitinase (PR-DUB) complex composed of the catalytic subunit BAP1 and its binding partner ASXL1 (35). We failed to detect 2A-DUB/Mysm1, USP16/Ubp-M and BAP1, which could be due to poor antibody performance. However, we found ASXL1 being present at all L3MBTL2 sites (Figure 7). Of particular note is that at several L3MBTL2 target promoters (*CXCL2*, *LOX*, *CXCL10* and *JAM*2), the ASXL1 level was slightly but reproducibly increased in cells expressing the L3MBTL2 K675/700R mutant. The correlation between reduced H2AK119ub1 levels and increased ASXL1 occupancy at these promoters may suggest that SUMOylation of L3MBTL2 negatively affects recruitment of PR-DUB.

### SUMOylation of L3MBTL2 functions in transcriptional repression of endogenous target genes

**Figure 8.**
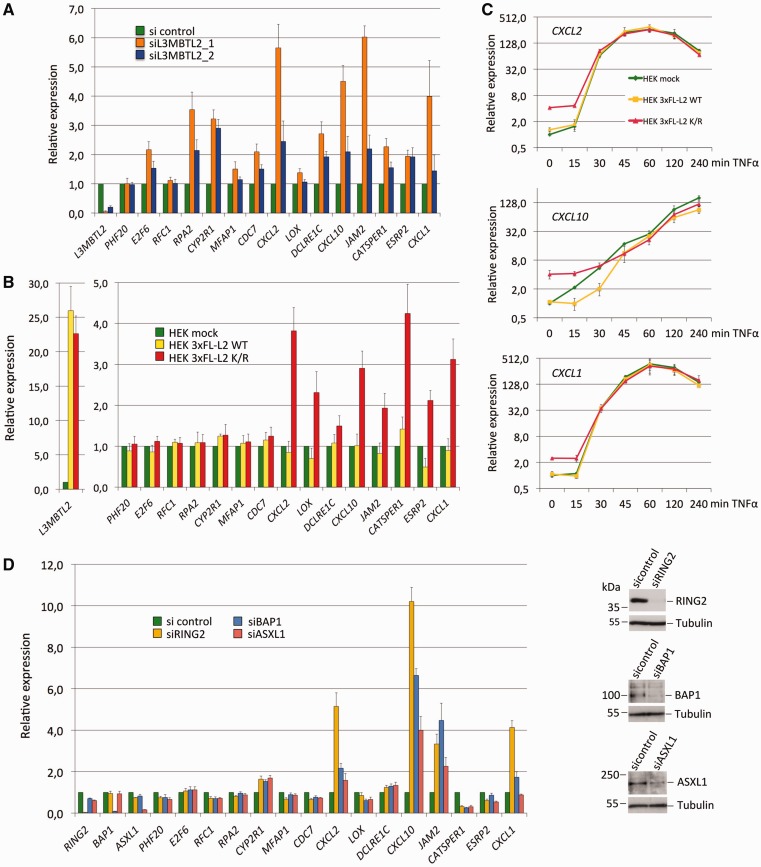
Expression of L3MBTL2 target genes. (**A**) HEK293 cells were transfected with either a control siRNA (green bars) or with two different siRNAs targeting *L3MBTL2* mRNA (orange and blue bars), and subsequently analysed for transcript levels of *L3MBTL2*, *PHF20*, *E2F6*, *RFC1*, *RPA2*, *CYPR1*, *MFAP1*, *CDC7*, *CXCL2*, *LOX*, *DCLRE1C*, *CXCL10*, *JAM2*, *CATSPER1*, *ESPR2* and *CXCL1. GAPDH* and/or *B2M (beta-2-microglobulin)* transcript levels were used for normalisation. Normalized transcript levels are presented relative to the control siRNA set to 1. Data are presented as the mean of three independent experiments ±SD. (**B**) Mock-transfected HEK293 cells (green bars) and HEK293 cells expressing either 3xFLAG-L3MBTL2 WT (yellow bars) or the 3xFLAG-L3MBTL2 K675/700R mutant (red bars) were analysed for L3MBTL2-target gene expression. Normalized transcript levels are presented relative to mock-transfected cells set to 1. Data are presented as the mean of three independent experiments ±SD. (**C**) Mock-transfected HEK293 cells (green lines) and HEK293 cells expressing either 3xFLAG-L3MBTL2 WT (yellow lines) or the 3xFLAG-L3MBTL2 K675/700R mutant (red lines) were treated with 20 ng/ml of TNFα for the indicated time periods, and subsequently analysed for *CXCL1*, *CXCL2* and *CXCL10* transcript levels. Normalized transcript levels are presented relative to untreated mock-transfected cells set to 1. Data are presented ±SD. (**D**) HEK293 cells were transfected with pools of siRNAs targeting *RING2, BAP1 or ASXL1* mRNA, and subsequently analysed for transcript levels of the indicated genes. *GAPDH* transcript levels were used for normalization. Data are presented as the mean of triplicates ±SD. The western blots on the right show reduction of RING2, ASXL1 and BAP1 protein levels upon siRNA treatment.

Given that SUMOylation of L3MBTL2 does not affect its recruitment to chromatin, we asked whether SUMOylation of L3MBTL2 affects target gene expression. At first we examined the transcriptional impact of endogenous L3MBTL2 in proliferating wild-type HEK293 cells by treating the cells with control or *L3MBTL2* siRNAs. Effective knockdown of L3MBTL2 was confirmed at the transcript (Figure 8A) and protein levels (Figure 1A). We then analysed the panel of target genes that was validated for L3MBTL2 occupancy. Upon knockdown of L3MBTL2, expression of eleven out of fifteen genes (*E2F6*, *RPA2*, *CYP2R1*, *CDC7*, *CXCL2*, *DCLRE1C*, *CXCL10*, *JAM2*, *CATSPER1*, *ESRP2* and *CXCL1*) increased by >2-fold (Figure 8A) suggesting that L3MBTL2 was functionally repressive at these promoters. Remarkably, none of the tested target genes showed reduced expression upon L3MBTL2 depletion. Next, we examined expression of these genes in cells expressing either wild-type FLAG-L3MBTL2 or the SUMOylation-defective FLAG-L3MBTL2 K675/700R mutant. Expression of *CXCL2*, *LOX*, *JAM2* and *ESRP2* was slightly reduced in cells expressing SUMOylated wild-type FLAG-L3MBTL2. In contrast, expression of these genes was significantly increased in cells expressing the SUMOylation-defective L3MBTL2 K675/700R mutant. Also expression of *DCLRE1C*, *CATSPER1* and *CXCL1*, which was not affected by over expression of wild-type L3MBTL2, showed increased expression in L3MBTL2 K675/700R mutant cells (Figure 8B). These results strongly suggest that SUMOylation of L3MBTL2 facilitates repression of endogenous target genes.

We realized that some of the genes that were occupied and repressed by L3MBTL2 such as *CXCL1*, *CXCL2* and *CXCL10* encode pro-inflammatory cytokines that are activated by inflammatory signals through the NFκB pathway. Therefore, we asked whether over expression of wild type FLAG-L3MBTL2 or the SUMOylation-defective L3MBTL2 K675/700 mutant would also affect the induction rate of these genes. Treatment of cells with TNFα rapidly induced expression of all three *CXCL* genes in wild-type HEK293 cells reaching a maximum by 1 h followed by a decline in the case of *CXCL1* and *CXCL2* (Figure 8C). The induction rates, the maximal expression and the decline of these cytokine genes in L3MBTL2 K675/700R expressing cells were similar as in wild-type HEK293 and in FLAG-L3MBTL2 WT expressing cells. This result suggests that SUMOylation of L3MBTL2 is essential for repression of the basal activity of these cytokine genes but not for limiting expression after activation.

At promoters that were affected by L3MBTL2 SUMOylation, de-repression correlated with reduced H2Aub1 levels. Therefore, we finally addressed whether repression of these L3MBTL2-target genes depends on the ubiquitinating RING2 enzyme and/or the de-ubiquitinating PR-DUB (BAP1/ASXL1) complex. Knockdown of *RING2* as well as knockdown of *BAP1* or *ASXL1* resulted in increased expression of *CXCL2*, *CXCL10*, *JAM2* and *CXCL1,* and decreased expression of *LOX, CATSPER1* and *ESRP2*, while expression of genes that were not affected by SUMOylation remained largely unchanged (Figure 8D). This result shows that RING2 as well as PR-DUB are functionally linked to the repression of genes that are regulated by L3MBTL2 SUMOylation. That depletion of the ubiquitinating as well as of the deubiquitinating enzyme results in de-repression matches the situation in *Drosophila*, where repression of a subset of PcG-target genes requires not only the H2A ubiquitinase activity of PRC1 but also the deubiquitinase activity of PR-DUB (35).

## DISCUSSION

In this study, we have unambiguously established the polycomb group protein L3MBTL2 as a target for SUMOylation. It is worthy of note that L3MBTL2 was also identified as a putative SUMO substrate in two previous proteomic studies (36,37). L3MBTL2 is specifically SUMOylated by SUMO2/3 at K675 and K700 close to the C-terminal end. SUMOylated endogenous L3MBTL2 is readily detectable by western blotting, which is to some extent exceptional given that in most cases the SUMO-modified protein fraction at the steady state is very small in relation to the total protein pool.

We have found that PIAS1 can act as a SUMO E3 ligase *in vitro*. However, RNAi-mediated depletion of PIAS1 did not reduce SUMOylation of endogenous L3MBTL2. It is conceivable that other PIAS family members could also catalyze SUMOylation of L3MBTL2 *in vivo*. The polycomb protein PC2 (CBX4) is another well-established SUMO E3 ligase (38,39) that stimulates SUMOylation of several repressive proteins including CtBP (38), DNMT3a (40) and CTCF (41). Since L3MBTL2 is also a polycomb group protein, an obvious question was whether PC2 would be an E3 ligase for L3MBTL2 SUMOylation. Co-transfection of L3MBTL2 along with PC2 did not result in increased SUMOylation of L3MBTL2 (data not shown). Thus, it remains unclear at this stage, which SUMO E3 ligase acts on L3MBTL2 *in vivo*.

The most conspicuous structural feature of L3MBTL2 is the 4xMBT repeat domain, which was shown to bind specifically to H4K20me1 and H4K20me2 peptides in their isolated form (8) as well as in the context of full-length L3MBTL2 (11). However, it was also reported that histone tail recognition of full-length L3MBTL2 was independent of histone lysine methylation (12). In accordance with the reports of Guo and Trojer (8,12), we found that the isolated 4xMBT-repeat domain bound preferentially to a H4K20 di-methylated peptide, whereas full-length L3MBTL2 bound equally well to unmodified and methylated histone H3 and H4 peptides (Figure 4). Efficient SUMOylation of recombinant full-length L3MBTL2 *in vitro* also allowed us to analyse whether SUMOylation of L3MBTL2 affects binding to histone tails. Unmodified as well as SUMOylated full-length L3MBTL2 bound equally effective to H3 and H4 peptides regardless of mono-, di- or tri-methylation at H3K9 or H4K20 (Figure 4B, C and D). Whether, SUMOylation of L3MBTL2 would affect binding to other methyl marks on histone tails or binding to other methylated proteins remains to be tested.

Our ChIP-Seq analyses identified >8000 high confident L3MBTL2-binding sites in HEK293 cells, preferentially around TSSs. Comparison with ChIP-Seq datasets for H3K4me3 and RNA polymerase II (32) revealed a high correlation of L3MBTL2 occupancy with H3K4me3 and promoter-enriched RNA polymerase II. Some overlap of L3MBTL2-binding sites with the H3K4me3 mark was also observed in K562 cells (12) and in mouse embryonic fibroblasts (14). H3K4me3 is highly predictive for an open chromatin structure characteristic for actively transcribed genes and for repressed genes that are poised for transcription (42,43) implying that L3MBTL2 is bound predominantly to active promoter regions. The concurrent enrichment of RNA polymerase II at L3MBTL2 occupied promoter regions might further indicate the presence of a paused RNA polymerase II complex.

L3MBTL2 is a subunit of the E2F6.com1 (13) and the non-canonical PRC1.6 complex (15). Both complexes contain the heterodimeric transcription factors E2F6/DP1 and MGA/MAX, the latter binding to E-box and T-box motifs (44). Our motif search at the peak centre of the L3MBTL2-binding sites did not detect a strong association with E2F6-binding sites but with the E-box (CACGTG), which might indicate recruitment of L3MBTL2 through binding of MGA/MAX to DNA.

The chromatin localization as well as the amount of chromatin-bound wild-type FLAG-L3MBTL2 or the FLAG-L3MBTL2 K675/700R mutant was similar to endogenous L3MBTL2 despite of an ∼25-fold over-expression of the tagged proteins (Figure 8B) indicating that L3MBTL2 recruitment is saturated at physiological conditions. Importantly, this observation also implies that the SUMOylation-defective L3MBTL2 mutant replaced endogenous SUMOylated L3MBTL2 in FLAG-L3MBTL2 K675/700R expressing cells. Hence, SUMOylation of L3MBTL2 does not affect recruitment of L3MBTL2 to its sites in chromatin. However, SUMOylation of L3MBTL2 appears to impact the repression of a subset of L3MBTL2-target genes suggesting that SUMOylation of L3MBTL2 acts downstream of chromatin recruitment. That SUMOylation does not affect recruitment but activity on a subset of promoters is reminiscent to the transcription factor Sp3. SUMOylation of Sp3 does not affect binding to target promoters but is essential for the repression of a subset of target genes (45).

Our ChIP experiments revealed that high and low levels of L3MBTL2 correlate with high and low levels of E2F6 and RING2, which is consistent with the presence of all three proteins in the PRC1L4/PRC1.6 complex (12,15). Surprisingly, however, the levels of L3MBTL2/E2F6/RING2 inversely correlated with the level of H2AK119ub1 (Figure 7). Most significantly, promoters with low levels of L3MBTL2/E2F6/RING2 contained high levels of H2AK119ub1 (Figure 7). This seems to be inconsistent as H2AK119 ubiquitination is catalysed by RING2, and L3MBTL2 is required to promote H2AK119 ubiquitination at several PRC1L4/PRC1.6 target genes in HEK293 cells (12). A likely explanation could be that high levels of H2AK119ub1 reflect a high local histone density (‘compacted chromatin’), which could result in reduced antibody accessibility of L3MBTL2, E2F6 and RING2. In line with this interpretation, low levels of L3MBTL2/E2F6/RING2 and high levels of H2AK119ub1 were found particularly at repressed genes whose basal expression is known to be very low such as cytokine genes in the absence of pro-inflammatory signals. Our expression analysis revealed that particularly these ‘low basal level genes’ were de-repressed when the SUMOylation-defective L3MBTL2 K675/700R mutant was bound suggesting that SUMOylation of L3MBTL2 facilitates repression of these genes. How SUMOylation acts mechanistically in this process remains elusive. We have found that de-repression of these genes was accompanied with slightly but significantly reduced H2AK119ub1 levels (Figure 7) suggesting that SUMOylation of L3MBTL2 promotes mono-ubiquitination or, alternatively, prevents deubiquitination of histone H2AK119. Occupancy of the H2A ubiquitinating enzyme RING2 was unchanged in FLAG-L3MBTL2 WT or FLAG-L3MBTL2 K675/700R over-expressing cells. However, we found that binding of ASXL1, the non-catalytic subunit of the dimeric polycomb repressive deubiquitinase PR-DUB (35) was slightly increased in cells expressing the SUMOylation-defective FLAG-L3MBTL2 K675/700R mutant (Figure 7) suggesting that SUMOylation of L3MBTL2 may impair recruitment of the PR–DUB complex. A functional link between RING2 and PR–DUB occupancy, and gene expression was established by RNAi experiments. Both, depletion of the H2A ubiquitinating enzyme RING2 as well as of the de-ubiquitinating PR–DUB resulted in de-repression of several genes that were regulated by SUMOylation of L3MBTL2. That depletion of counteracting H2A-ubiqitinating and deubiquitinating enzymes resulted in de-repression may seem paradoxical; however, it coincides with observations in *Drosophila* (35,46). Apparently, appropriately balanced H2Aub1 levels are critical for maintaining the repressed state of a subset of L3MBTL2 target genes. Thus, it could be proposed that SUMOylation of L3MBTL2 facilitates repression of a subset of target genes by fine-tuning local H2Aub1 levels.

## SUPPLEMENTARY DATA

Supplementary Data are available at NAR Online.

Supplementary Data
