# Supplementary material for: SUMOylation of the polycomb group protein L3MBTL2 facilitates repression of its target genes
Source: Nucleic Acids Res. 2013 Dec 24;42(5):3044–58. doi: 10.1093/nar/gkt1317 (PMC3950706; doi:10.1093/nar/gkt1317)
Supplement: Supplementary Data [file supp_42_5_3044__index.html]

SUMOylation of the polycomb group protein L3MBTL2 facilitates repression of its target genes — Supplementary Data 

# SUMOylation of the polycomb group protein L3MBTL2 facilitates repression of its target genes

## Supplementary Data

files

**Files in this Data Supplement:**

- Supplementary Data - doc file
- Supplementary Data - xls file
