# Supplementary material for: SUMOylation of the polycomb group protein L3MBTL2 facilitates repression of its target genes
Source: Nucleic Acids Res. 2013 Dec 24;42(5):3044–58. doi: 10.1093/nar/gkt1317 (PMC3950706; doi:10.1093/nar/gkt1317)
Supplement: Supplementary Data [file supp_gkt1317_nar-01829-x-2013-File010.doc]

**Supplementary Data (Stielow *et al.*)**

**Supplementary Materials and Methods**

**Antibodies**

For immunodetection and ChIP experiments, the following antibodies were used: anti-L3MBTL2 (Active Motif, 39569), anti-Tubulin (Millipore, MAB3408), anti-FLAG M2 (Sigma, F3165), anti-5xHis (Qiagen, 34660), anti-GAL4 (Covance, PRB, 255C), anti-RING2 (Abcam, Ab101273), anti-E2F6 (Santa Cruz, sc-22823), anti-Ubiquityl-Histone H2A (Lys119) (Cell Signaling, 8240), anti-BAP1 (Santa Cruz, sc-13576), anti-ASXL1 (Santa Cruz, sc-98302) and anti PIAS1 (Abcam, ab77231). The SUMO1 antibody and the SUMO2/3 antibody were gifts from M. Matunis.

Primers for ChIP-qPCR analyses

| **Promoter** | **Forward Primer 5´-3´** | **Reverse Primer 5´-3´** |
| --- | --- | --- |
| *PHF20* | tgagtggggacttcgtgttc | gaccaaccgacagaaggact |
| *E2F6* | cctgttcccttcctctggaa | cgacgcagacggaaaaagag |
| *RFC1* | gccaaaaaccgagctcacac | ccattcgcgccaacaacttc |
| *RPA2* | cacgccgaacaaaggaagtg | cagttggctccaaaagcctc |
| *CYP2R1* | tctcagccgcgcaaaactag | gccctcttcagctctccaaa |
| *MFAP1* | tcattcgaaccttcctcacc | aaccggaaatacgtgacgag |
| *CDC7* | gagccacagaagtcgtactc | ccgaaccagatgcttagtgc |
| *CXCL2* | taagggatctgacccacgac | agttcggaaggaaggcgatg |
| *LOX* | taacgctccctgtgcaacgt | cctttcccctttctcagtcc |
| *DCLRE1C* | ccggatgctccttgactttg | accccaaaaccgcagctgaa |
| *CXCL10* | ccttcgagtctgcaacatgg | gttcctctgctgtaggctca |
| *JAM2* | tccacccctaggctgaaaag | gatcggctttgtgtctggtc |
| *CATSPER1* | gggcggtttataggactcag | agccttccccgaccttacat |
| *ESRP2* | gaccagccctggaagtagga | ctctccaggcgagttacctg |
| *CXCL1* | tggggcagaaggcgaatatc | tccgcgaaccccttttatgc |
| *CDC7 -2kb* | caccttcttacctcacagac | gggtatagttcagggtgaag |
| *UGGT2* | ccaaagtcctgggaatggac | agaaaagcgcgagtccctcg |
| *PRAF2* | catctcgcttcgtggcaaag | cgacatcctgccggttaatg |
| *DENND3* | ctagaaaataccgacgggcc | cgctatcatggccacagttg |

**Primers for RT-qPCR analyses**

| **Gene** | **Forward Primer 5´-3´** | **Reverse Primer 5´-3´** |
| --- | --- | --- |
| *L3MBTL2* | aggccaaagaggccacaaag | gccacgtctagatgctcttc |
| *PHF20* | ctccaaaaaggccctaccag | tagtccagccagctctccaa |
| *E2F6* | tggatgttccagctcccaga | ttcttcctcagggccttctg |
| *RFC1* | ggtggcaaacctagtccctt | attccgaatccagggatggg |
| *RPA2* | ccaggaatgagtgaagcagg | tcaggtacccagttagatcca |
| *CYP2R1* | gcgatttctggacagcagtg | tcatgcctaacctgggcttc |
| *MFAP1* | ttcgggcaaacggcaaagtc | gacctgagcgtccaaagttc |
| *CDC7* | acatgtggtctgcaggtgtc | tgggagtgctagaatccatac |
| *CXCL2* | cctgcagggaattcacctca | ccttccttctggtcagttgg |
| *LOX* | cagtgtaaaccccagctacc | cttcagaacaccaggcactg |
| *DCLRE1C* | agcctttatgccggtcttcc | cagcatcctggggtttgtct |
| *CXCL10* | gaaagcagttagcaaggaaaggt | gacatatactccatgtagggaag |
| *JAM2* | ccgtttgtggccttggtgta | ttccatttcctcgtgtacctc |
| *CATSPER1* | atgctgaagcggctcatcga | ggggtcaattcctgaagtcc |
| *ESRP2* | agatgagccgagtgctgatg | cagtgggtgtagtgaggtag |
| *CXCL1* | tcctgcatcccccatagtta | cttcaggaacagccaccagt |
| *RING2* | ggcctcatcccacacttatg | tggccactggctgttgctat |
| *ASXL1* | cgaaaagccacagcccacta | gggggcatatctggtaagtg |
| *BAP1* | cctcaaggaggaggtagaga | gttctgctccactaggttgg |
| *GAPDH* | tgcaccaccaactgcttagc | ggcatggactgtggtcatga |
| *B2M* | agtatgcctgccgtgtgaac | ggagcaacctgctcagatac |

**Plasmids**

The expression vector for L3MBTL2-FLAG (pCMV-tag4A-L3MBTL2) has been described in . The corresponding point mutants (K541R, K675R, K700R, K675/700R, K541/675/700R) were generated using either the QuikChange XL Site-Directed Mutagenesis Kit or the QuikChange Multi Site-Directed Mutagenesis Kit (both Stratagene) according to the manufacturer´s instructions. Expression constructs for SUMO1, 6xHis-SUMO1 and 6xHis-SUMO2 (pSG-SUMO1, pSG-His-SUMO1 and pSG-His-SUMO2) were kindly provided by S. Müller. Vectors for the expression of siRNAs targeting SUMO2 and SUMO3 were generated by cloning appropriate BglII/HindIII-flanked oligonucleotides into pTer . The plasmid expressing the SUMO1 siRNA (pSuper.retro-siRNA-SUMO1) was a kind gift of R. Hay. The expression vector for the 6xHis-tagged three MBT domains of L3MBTL1 (pASK43-3MBT) has been described previously . The construct expressing recombinant L3MBTL2 (pASK43-L3MBTL2) was kindly provided by P. Trojer and D. Reinberg. 6xHis-L3MBTL2K675/700R and the four MBT domains of L3MBTL2 (6xHis-4xMBT) were isolated as BamHI/XhoI fragments from the pCMV-tag4A-L2flK675/700R and pCMV-tag4A-L3MBTL2 plasmids, respectively, and subsequently cloned into pASK43. The GAL4-dependent firefly reporter plasmid p(UAS)4xtk-Luc was kindly provided by A. Baniahmad and R. Renkawitz. The SV40 promoter-driven *Renilla* co-reporter is commercially available (Promega). The constructs expressing GAL4-L3MBTL1 as well as wild type and mutant GAL4-L3MBTL2 were generated by inserting the cDNA of either L3MBTL1, L3MBTL2 or L3MBTL2 mutants as HindIII/BamHI fragments into pGAL4. The plasmids encoding 3xFLAG-L3MBTL2 (pN3-3xFLAG-L3MBTL2) and the corresponding SUMOylation-deficient mutant (pN3-3xFLAG-L3MBTL2K675/700R) were obtained by re-cloning appropriate fragments from pCMV-tag4A-L3MBTL2 and pCMV-tag4A-L2flK675/700R into the CMV promoter-driven vector pN3-3xFLAG. The parental plasmid pN3-3xFLAG was generated by inserting a hybridized 3xFLAG oligonucleotide as BglII/HindIII fragment into pN3.

**Reporter gene assay**

HEK293 cells were seeded on 24-well plates (6x10^4^ cells/well). Upon 24 hours cells were transfected with 200 ng of a GAL4-dependent firefly-luciferase reporter plasmid (p(UAS)4xtk-Luc), 250 ng of plasmids expressing either GAL4, GAL4-L3MBTL1, GAL4-L3MBTL2 or GAL4-L3MBTL2 mutants and 0.5 ng of the *Renilla* luciferase plasmid pRL-SV40 (Promega) as internal reference. Forty-eight hours post transfection, cells were lysed and firefly as well as Renilla luciferase activities were determined using the Dual Luciferase Kit (Promega) and the Berthold AutoLumat Plus LB953 multi-tube luminometer.

**Stable cell lines**

For stable expression of either 3xFLAG-L3MBTL2 or 3xFLAG-L3MBTL2 K675/700R, 1.4x10^6^ HEK293 cells having a stably integrated GAL4-responsive luciferase reporter were transfected with 1 µg of linearized pN3-3xFLAG-L3MBTL2 or pN3-3xFLAG-L3MBTL2 K675/700R, respectively. Forty-eight hours upon transfection cells were selected with 1 µg/ml puromycin and 1 µg/µl G418. Single clones were isolated, expanded and L3MBTL2 expression was analyzed by western blotting.

**Western Blotting**

For analysis of L3MBTL2 SUMOylation, peptide binding and immunoprecipitation proteins were separated by SDS-PAGE and subsequently transferred to an Immobilon-P membrane (Millipore) according to the manufacturer´s instructions. Upon blocking in 5% skimmed milk primary antibody incubations were carried out in 1% skimmed milk for one hour at room temperature or over night at 4 °C. Incubations with HRP-coupled secondary antibodies were performed in 1% skimmed milk for one hour at room temperature. Proteins were visualized by incubation with the Immobilon HRP-substrate Western Detection solution (Millipore).

**Immunoprecipitation of 3xFLAG-L3MBTL2 or 3xFLAG-L3MBTL2 K675/700R**

Nuclear extracts from HEK293 cells stably expressing either 3xFLAG-L3MBTL2 or 3xFLAG-L3MBTL2 K675/700R were prepared essentially according to with the exception that all buffers contained 20 mM N-ethylmaleimide (NEM) to sustain proteome-wide SUMOylation. Prior to antibody incubation, NEM was removed by dialysis. In brief, 750 µg of nuclear extract were dialysed against IP buffer (25 mM HEPES/KOH, pH 7.9, 12.5 mM MgCl_2_, 150 mM NaCl, 0.1 mM EDTA, 10% glycerol, 0.1% NP 40, 1 mM PMSF, 0.5x PIC). Extracts were precleared for 90 minutes at 4 °C on a rotating wheel using 20 µl of Mouse IgG-agarose (Sigma). The cleared supernatant was incubated with 20 µl of anti-FLAG M2 affinity gel (Sigma) for three hours at 4 °C on a rotating wheel. Beads were collected by centrifugation and washed five times in washing buffer (25 mM HEPES/KOH, pH 7.9, 12.5 mM MgCl_2_, 300 mM NaCl, 0.1 mM EDTA, 10% glycerol, 0.08% NP 40, 1 mM PMSF, 0.5x PIC). Bound proteins were eluted five times each with 2 µg of 3xFLAG peptide for 30 minutes at 1200 rpm and 4 °C and eluates were pooled.

**Quantitative real-time PCR**

Two µg of total RNA was used for cDNA synthesis with the SuperScript II Reverse Transcriptase kit (Invitrogen) using 0.5 µg oligo(dT) as priμer according to the manufacturer’s instruction. Quantitative RT-PCR was performed in triplicate with 1 µl of 1:20 diluted cDNA and gene-specific primers on the Mx3000P real-time PCR system (Stratagene) using ImmoMix^TM^ (Bioline) and SYBR Green (Invitrogen). Values were normalized to *GAPDH and/or B2M* mRNA content.

**Supplementary Table S1.** List of gene abbreviations.

| **Gene symbol** | **Gene full name** |
| --- | --- |
| *L3MBTL2* | Lethal(3)malignant brain tumor-like 2 |
| *PHF20* | PHD finger protein 20 |
| *E2F6* | E2F transcription factor 6 |
| *RFC1* | Replication factor C 1 |
| *RPA2* | Replication protein A 2 |
| *CYP2R1* | Cytochrome P450, family 2, subfamily R1 |
| *MFAP1* | Microfibrillar-associated protein 1 |
| *CDC7* | Cell division cycle 7 |
| *CXCL2* | Chemokine (C-X-C motif) ligand 2 |
| *LOX* | Lysyl oxidase |
| *DCLRE1C* | DNA cross-link repair 1C |
| *CXCL10* | Chemokine (C-X-C motif) ligand 10 |
| *JAM2* | Junctional adhesion molecule 2 |
| *CATSPER1* | Cation channel, sperm associated 1 |
| *ESRP2* | Epithelial splicing regulatory protein 2 |
| *CXCL1* | Chemokine (C-X-C motif) ligand 1 |
| *UGGT2* | UDP-glucose glycoprotein glucosyltransferase 2 |
| *PRAF2* | PRA1 domain family, member 2 |
| *DENND3* | DENN/MADD domain containing 3 |
| *ASXL1* | Additional sex comb like 1 |
| *BAP1* | BRCA1 associated protein |
| *GAPDH* | Glyceraldehyde-3-phosphate dehydrogenase |
| *B2M* | Beta-2 microglobulin |

**Supplementary Figures**


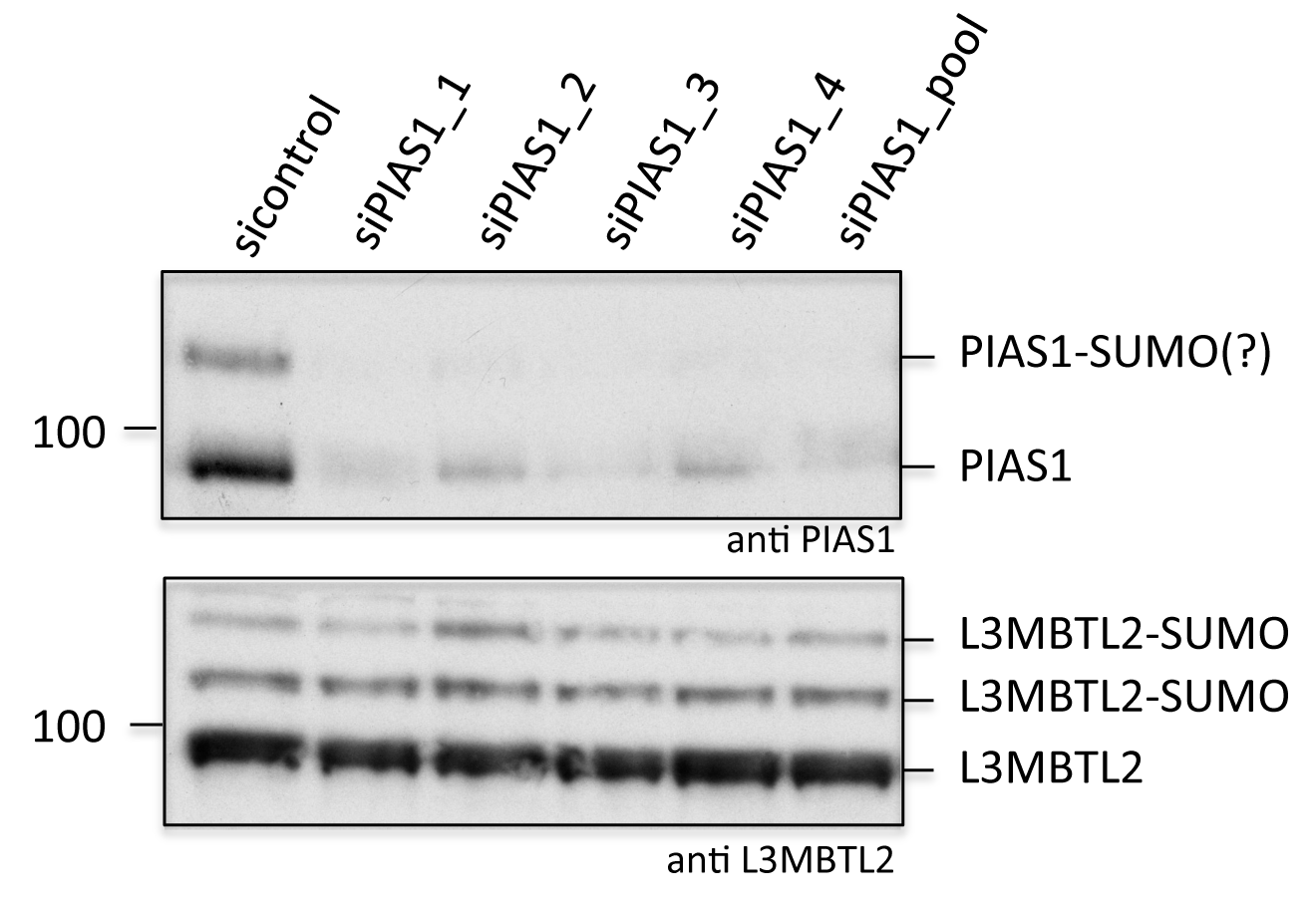


**Supplementary Figure S1**: HEK293 cells were transfected with four different siRNAs or a pool thereof targeting *PIAS1.* Subsequently, whole cell extracts were analysed for PIAS1 and L3MBTL2 protein levels by western blotting.

**
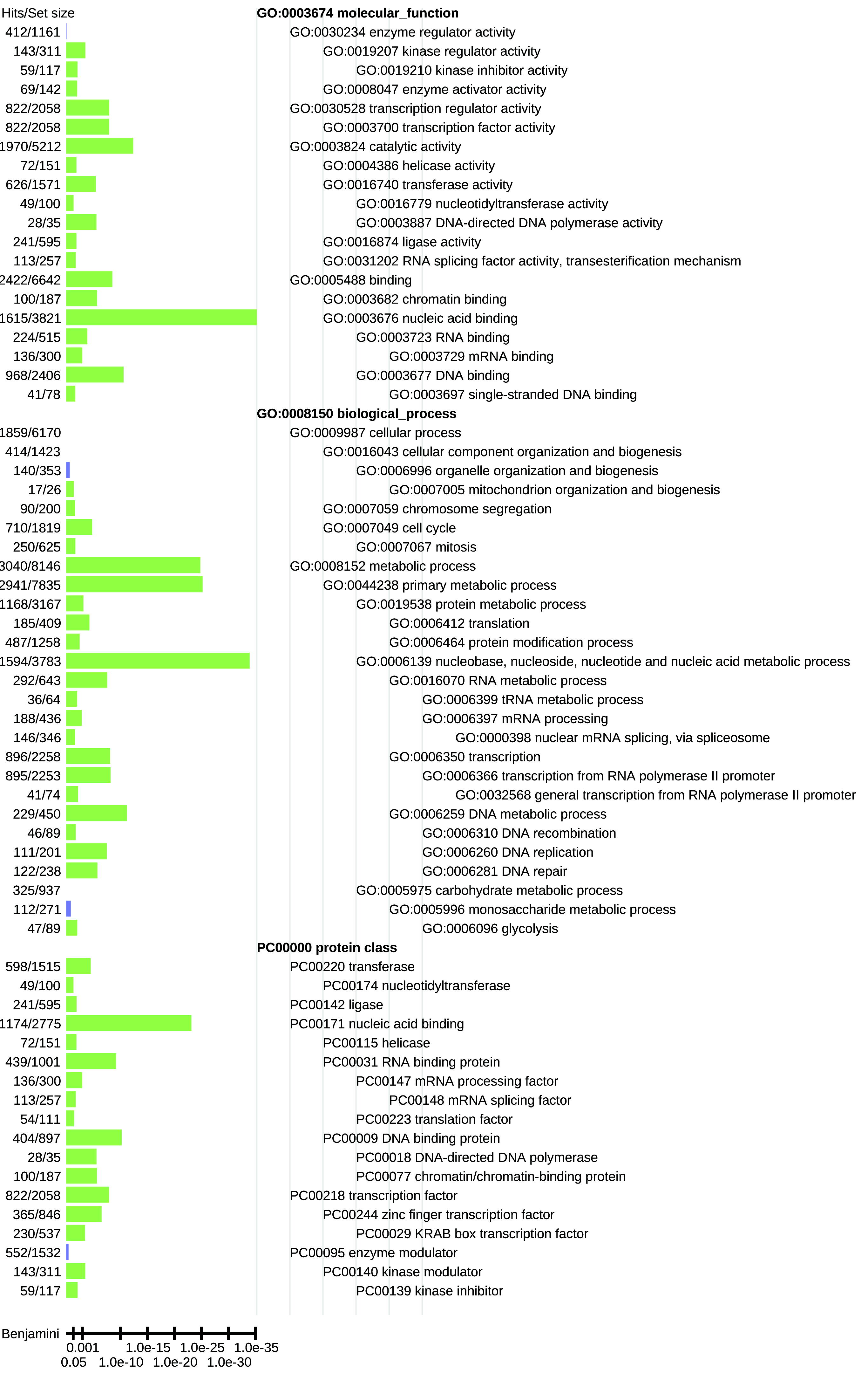
**

**Supplementary Figure S2.** Enriched PANTHER terms for 4587 annotated L3MBTL2 target genes. The number of hits per set size is shown on the left side. Bars are drawn proportional to -log_10_ (Benjamini-value). Indentions indicate hierarchies in PANTHER annotations. P-value for intersection between PANTHER 7.0-derived gene sets and the L3MBTL2 set were calculated via Fisher's exact test and corrected with the Benjamini-Hochberg procedure.

**
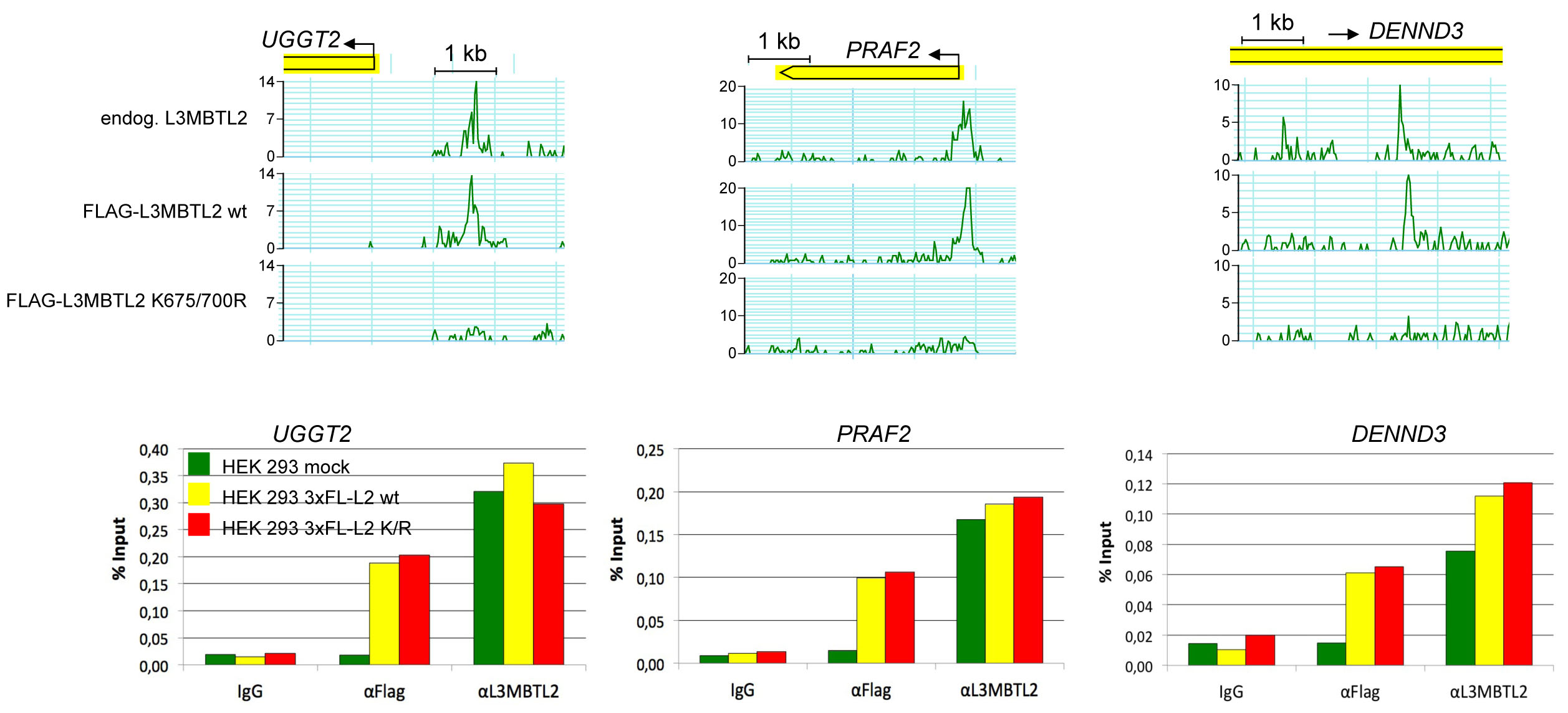
**

**Supplementary Figure S3.** Top, genome browser snapshots of ChIP-Seq binding patterns of endogenous L3MBTL2 and ectopically expressed 3xFLAG L3MBTL2 wt and 3xFLAG L3MBTL2 K675/700R. Bottom, ChIP-qPCR analysis of L3MBTL2 occupancy at the ChIP-Seq peaks.

**Supplementary Dataset 1 (see separate excel file)**

16,569 L3MBTL2, FLAG-L3MBTL2 and FLAG-L3MBTL2 K675/700 peaks as determined by ChIP-Seq.

Columns:

A: chromosome

B and C: chromosomal region spanning the L3MBTL2 peak

D: Distance of peak to next TSS

E and F: Name and Ensemble ID of the next gene

G: Gene description

H: FDR of endogenous L3MBTL2 peak

I: FDR of 3xFLAG-L3MBTL2 WT peak

J: FDR of 3x FLAG-L3MBTL2 K675/700R peak

K: Position relative to next gene

L: Tag count of endogenous anti L3MBTL2 peak

M: Tag count of endogenous anti L3MBTL2 peak normalized to 10 million reads

N: Tag count of anti FLAG-L3MBTL2 WT peak

O: Tag count of anti FLAG-L3MBTL2 WT peak normalized to 10 million reads

P: Tag count of anti FLAG-L3MBTL2 K675/700R peak

Q: Tag count of anti FLAG-L3MBTL2 K675/700R peak normalized to 10 million reads

R: Tag count of anti FLAG control in WT HEK293 cells

S: Tag count of anti FLAG control in WT HEK293 cells normalized to 10 million reads

T: Tag count of IgG control in WT HEK293 cells

U: Tag count of IgG control in WT HEK293 cells normalized to 10 million reads

The table is sorted by column M: Tag count of endogenous anti L3MBTL2 peak normalized to 10 million reads

**References to Supplementary Data**

Andrews, N.C., and Faller, D.V. (1991). A rapid micropreparation technique for extraction of DNA-binding proteins from limiting numbers of mammalian cells. Nucleic Acids Res *19*, 2499.

Matunis, M.J., Coutavas, E., and Blobel, G. (1996). A novel ubiquitin-like modification modulates the partitioning of the Ran-GTPase-activating protein RanGAP1 between the cytosol and the nuclear pore complex. J Cell Biol *135*, 1457-1470.

Stielow, B., Sapetschnig, A., Wink, C., Krüger, I., and Suske, G. (2008). SUMO-modified Sp3 represses transcription by provoking local heterochromatic gene silencing. EMBO Rep *9*, 899-906.

Trojer, P., Cao, A.R., Gao, Z., Li, Y., Zhang, J., Xu, X., Li, G., Losson, R., Erdjument-Bromage, H., Tempst, P.*, et al.* (2011). L3MBTL2 protein acts in concert with PcG protein-mediated monoubiquitination of H2A to establish a repressive chromatin structure. Mol Cell *42*, 438-450.

Trojer, P., Li, G., Sims, R.J., 3rd, Vaquero, A., Kalakonda, N., Boccuni, P., Lee, D., Erdjument-Bromage, H., Tempst, P., Nimer, S.D.*, et al.* (2007). L3MBTL1, a histone-methylation-dependent chromatin lock. Cell *129*, 915-928.

van de Wetering, M., Oving, I., Muncan, V., Pon Fong, M.T., Brantjes, H., van Leenen, D., Holstege, F.C., Brummelkamp, T.R., Agami, R., and Clevers, H. (2003). Specific inhibition of gene expression using a stably integrated, inducible small-interfering-RNA vector. EMBO Rep *4*, 609-615.

Zhang, X.D., Goeres, J., Zhang, H., Yen, T.J., Porter, A.C., and Matunis, M.J. (2008). SUMO-2/3 modification and binding regulate the association of CENP-E with kinetochores and progression through mitosis. Mol Cell *29*, 729-741.
